# Mosaic structure of intragenic repetitive elements in histone H1-like protein Hc2 varies within serovars of *Chlamydia trachomatis*

**DOI:** 10.1186/1471-2180-10-81

**Published:** 2010-03-17

**Authors:** Markus Klint, Mikael Thollesson, Erik Bongcam-Rudloff, Svend Birkelund, Anders Nilsson, Björn Herrmann

**Affiliations:** 1 Department of Clinical Microbiology, Uppsala University, Uppsala, Sweden; 2 Department of Evolution, Genomics and Systematics, Uppsala University, Uppsala, Sweden; 3 Linnaeus Centre for Bioinformatics, Uppsala University, Uppsala, Sweden; 4 Department of Medical Microbiology and Immunology, Aarhus University, Aarhus, Denmark

## Abstract

**Background:**

The histone-like protein Hc2 binds DNA in *Chlamydia trachomatis *and is known to vary in size between 165 and 237 amino acids, which is caused by different numbers of lysine-rich pentamers. A more complex structure was seen in this study when sequences from 378 specimens covering the *hctB *gene, which encodes Hc2, were compared.

**Results:**

This study shows that the size variation is due to different numbers of 36-amino acid long repetitive elements built up of five pentamers and one hexamer. Deletions and amino acid substitutions result in 14 variants of repetitive elements and these elements are combined into 22 configurations. A protein with similar structure has been described in *Bordetella *but was now also found in other genera, including *Burkholderia*, *Herminiimonas*, *Minibacterium *and *Ralstonia*.

Sequence determination resulted in 41 *hctB *variants that formed four clades in phylogenetic analysis. Strains causing the eye disease trachoma and strains causing invasive lymphogranuloma venereum infections formed separate clades, while strains from urogenital infections were more heterogeneous. Three cases of recombination were identified. The size variation of Hc2 has previously been attributed to deletions of pentamers but we show that the structure is more complex with both duplication and deletions of 36-amino acid long elements.

**Conclusions:**

The polymorphisms in Hc2 need to be further investigated in experimental studies since DNA binding is essential for the unique biphasic life cycle of the *Chlamydiacae*. The high sequence variation in the corresponding *hctB *gene enables phylogenetic analysis and provides a suitable target for the genotyping of *C. trachomatis*.

## Background

*Chlamydia trachomatis *is an intracellular bacterium that can multiply only within a host cell. Reticulate bodies (RBs) are the replicating form of *Chlamydia *bacteria that transform into infectious elementary bodies (EBs) prior to cell lysis. The transformation from a fragile RB to a robust EB is dramatic: the size reduces from 1 μm to 0.3 μm, the dispersed chromatin aggregates into a condensed nucleoid and metabolism ceases.

*C. trachomatis *is classified into 19 serotypes (A-L3) based on the major outer membrane protein (MOMP) where A-C cause trachoma, D-K cause urogenital infections and L1-L3 cause lymphogranuloma venereum (LGV).

Hc1 and Hc2 are DNA-binding proteins, homologous to eukaryotic histone H1 [1,2] which are thought to mediate the chromatin compaction. These histone-like proteins are encoded by the *hctA *and *hctB *genes that are expressed late in the life cycle when RBs convert to EBs [3]. The *hctA *gene has been inserted into *Escherichia coli *and the expressed Hc1 was shown to induce a compaction of chromatin into a spherical condensed nucleoid [4]. Hc2 also condenses DNA but the nucleoid is distinctly different with a more thoroid shape [5,6], indicating that these proteins interact with DNA in different ways. Both proteins are able to repress transcription, but Hc2 has a higher binding affinity for RNA and thus represses translation more efficiently than Hc1 [6].

The Hc1 protein has two domains: the conserved N-terminus [7], which mediates dimerisation, and the lysine-rich C-terminus, which is responsible for DNA binding [8,9]. Hc2, on the other hand, varies in size between serovars because of varying numbers of lysine-rich pentameric repeats [10]. Hc2 appears to be ubiquitous in *Chlamydiaceae *because the *hctB *gene has been found in all available genome sequences of this family, Based on Southwestern blot analysis, Hc2 has previously been reported to be absent or present in reduced amounts in *Chlamydophila psittaci *strain Mn [10]. However, the *hctB *gene has been found in *C. psittaci *strain 6BC by whole genome sequencing (G. Myers, personal communication).

The *hctB *gene encoding Hc2 is one of the targets in a newly developed multilocus sequence typing (MLST) system for *C. trachomatis *[11]. Studies of trachoma, lymphogranuloma venereum (B. Herrmann, unpublished), the Swedish variant with plasmid mutated strains [12] and other genotyping studies have used this MLST system and the corresponding *hctB *sequences are stored in a publically-accessible MLST database [13].

When comparing *hctB *sequences from many *C. trachomatis *specimens it was clear that the size variation was more complex than could be attributed to simple deletions of a pentamer as previously described. In this study we found elements of 108 bp that are deleted and duplicated within the *hctB *gene without a premature stop codon or loss of the reading frame. We have created a nomenclature to characterise the variation in numbers and type of these elements observed in 378 clinically derived and reference specimens of *C. trachomatis*.

## Results

### Hc2 in *C. trachomatis*

**Figure 1:**
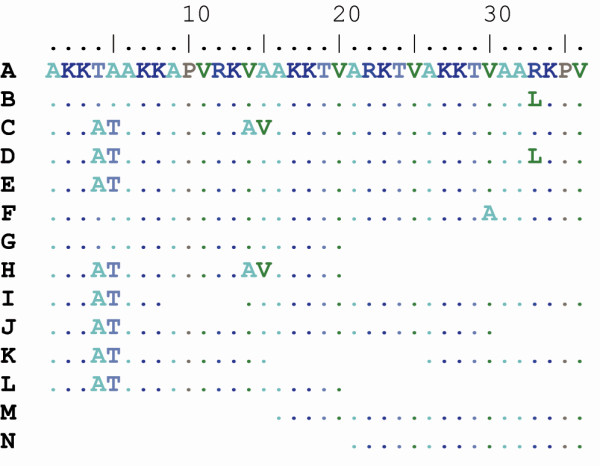
**Amino acid alignment of the 14 variants of repetitive elements (A-M) found in Hc2 of *Chlamydia trachomatis *among 378 specimens in the MLST database**.

**Figure 2:**
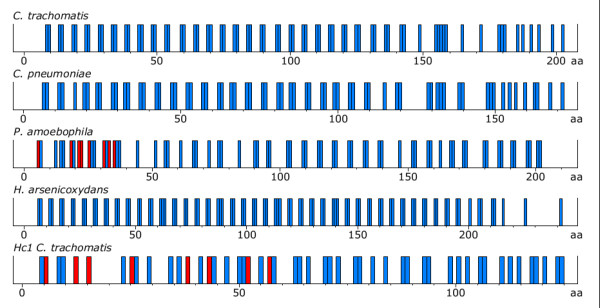
**Charge distribution in Hc2, Hc2-like proteins and Hc1**. Positively charged residues (blue bars) and negatively charged residues (red bars) in the protein sequence of Hc2 in *Chlamydia trachomatis*, *Chlamydophila pneumoniae*, *Protochlamydia amoebophila*, an Hc2-like protein in *Herminiimonas arsenicoxydans *and Hc1 in *Chlamydia trachomatis*.

41 *hctB *gene variants were found among 378 sequences in the MLST database, with the highest level of variation occurring in a region encoding consecutive amino acid pentamers. The pentamers have two positively charged residues (arginine and lysine) and three other residues that are mainly alanine, but also valine, threonine and proline (Figure 1). The pentamers result in evenly distributed positive charges throughout the Hc2 protein, except for the C-terminal domain (Figure 2). This charge distribution is in contrast to the DNA-binding C-terminal domain of Hc1 that has a random distribution of positive charges. The C-terminal domain of both Hc1 and Hc2 lack negatively charged residues.

Analysis of the amino acid sequence revealed that there was a repetitive structure within Hc2, with repetitive elements of 36 amino acids built up by six pentamers and one hexamer (Figure 1). The repetitive region in Hc2 is 72-144 amino acids long and has from two to four repetitive elements. Repetitive elements with deletions of 1-4 hexamer/pentamers are relatively rare though elements of 16, 20, 21, 26, 30 and 31 amino acids have been found.

**Figure 3:**
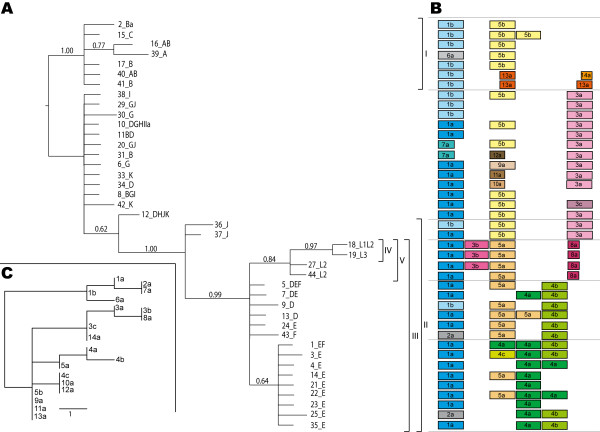
**Phylogenetic relationships of 41 variants of the MLST target that include *hctB *from *Chlamydia trachomatis***. (A) Phylogenetic tree based on the MLST target that includes the *hctB *gene. Each variant of the MLST target is indicated by the allele number and the serotypes in which that variant has been found. The phylogeny has been estimated using Bayesian inferences and rooted using paralog rooting based on the repetitive elements. The numbers on branches are posterior probabilities. The clades discussed in the text have been designated I-V. The repetitive elements found in each MLST variant are illustrated in an alignment to the right (B). The alignment of the repetitive elements is based on the neighbor-joining phylogeny of the element types (C) where the scale bar represents one nucleotide change.

A nomenclature was devised that enabled classification of the repetitive elements into 14 groups (denoted 1-14) based on the protein sequence (Figure 1) and 20 subgroups (1a, 1b, 2a etc) based on silent substitutions at the nucleotide level. There are 22 combinations of repetitive elements at the protein level (i.e. 1, 5 and 1, 5, 5) and 30 configurations at the nucleotide level (i.e. 1b, 5b and 1b, 5b, 5b) of Hc2 based on the 378 specimens in the MLST database (Figure 3). The 1, 2, 6 and 7 elements have a characteristic alanine-threonine instead of threonine-alanine in the first pentamer (Figure 1) compared with other elements, and they are always first of the elements in the repetitive region. The other major types of repetitive elements are 3, 4 and 5 that are separated by three amino acid substitutions. The 8-14 elements are shorter forms of 3, 4 and 5 with deletions of 5 to 20 amino acids.

The amino acid sequence outside the variable region is highly conserved with no insertions or deletions. The beginning of the gene encodes 24 amino acids with two substitutions; one of these substitutions is restricted to the B (genital), D, G, H, I, Ia, J and K serovars while the other is found in some trachoma strains. The last 69 amino acids of Hc2 downstream of the variable region are therefore partly excluded in MLST typing analysis. The only differences in sequence found in the 87 bp obtained with MLST sequencing are two substitutions that both cause a change in amino acid. One substitution was unique for the D, G, H, J and K serovars and one was found only in a trachoma strain. Additional sequencing was done in order to cover the last 120 bp of the *hctB *gene for 17 strains representing different types of Hc2. Only three variable positions were found. Two substitutions, of which one is silent, separate the LGV serovars from the others and one silent substitution is unique for the D, G, H, J and K serovars.

### Phylogeny and evolution of repeat elements

The phylogenetic analyses of the repeat elements (Figure 3C) and of the MLST target including *hctB *(Figure 3A), together show that the evolution of the *hctB *variants is characterized by a relatively rapid rate of within-genome duplications and deletions of repeat elements and a relatively slow rate of nucleotide substitution.

The phylogenetic tree shows that the *hctB *gene variants cluster in agreement with disease causing properties. The 41 variants of *hctB *sequences obtained with MLST gave a topology with posterior probability above 0.95 for four clades, designated I-IV (Figure 3). Clade I (1.0 posterior probability) contained the trachoma serovar A, B and C strains, but not the genital serovar B (alleles 8_BGI, 11_BD and 31_B). The serovars B (genital), D (D/UW3), G, H, I, Ia, J and K were part of an unresolved, basal, grade. Clade III comprised, in addition to the LGV serovars, serovar D (D/IC-Cal8), E and F. Clade IV (pp 0.97) consisted of some of the LGV serovars. The overlapping clade V included all LGV serovars but did not have significant support (pp 0.84).

Three cases of possible recombination were identified, resulting in four recombined sequences (data not shown). The sequences with a possible recombined origin are 36_J, 37_J (same event), 12_DHJK and 30_G. Removing these sequences from the dataset before Bayesian analysis gave the same overall topology (data not shown), but with an increased number of clades with significant support.

The phylogenetic analysis of the repeat element types (Figure 3C) indicated a duplication in the ancestor to *C. trachomatis*, one copy resulting in the 1, 2, 6 and 7 group and the other in the group comprising the element types 3-5 and 8-14. Because the 1, 2, 6 and 7 elements are always found one per sequence and first in order, the structure can be described as 1 + 1-3 elements rather than 2-4. Mapping this pattern on the *hctB *phylogeny, the first element (1, 2, 6 and 7 super group) appeared to have evolved by substitutions and deletions only. The 2 element for example can have evolved through a series of nucleotide substitutions, or by deletion of the end of a 1 element and the beginning of a 4 element. The remaining elements (3-5 and 8-14 super group) appear to have a much higher rate of duplications and extinction of entire elements. Thus in a duplication of a 5b element one copy gave rise to the 3 group lineage and the other copy to 5a and subsequently to the 4 group lineage of elements, with later duplications and extinctions within both these lineages.

## Discussion

### Hc2 diversity in *C. trachomatis*

Hc2 displays considerable diversity in length and in sequence when comparing 378 *C. trachomatis *specimens. Sequence comparisons show that Hc2 is a highly structured protein with consecutive pentamers but also with repetitions of larger elements built up by six pentamers and one hexamer. These repeated elements were found in 14 amino acid variants combined differently resulting in 20 configurations and 11 length variants of Hc2.

The rearrangement of repetitive elements appears to be continuous in *C. trachomatis *because there are specimens with different configurations of repetitive elements but with identical *ompA *genotype and MLST profile. The diversity generated by several deletions and duplications while the flanking regions remain intact suggests that the Hc2 protein is vital for *Chlamydia*, and that the number of repetitions in the DNA-binding region has an important role for the organism. It is difficult to link the length of Hc2 to particular characteristics because many specimens in the MLST database lack additional information such as clinical manifestations and phenotypic differences. This needs further exploration. However, the size variation enabled by rearrangement of the repeated elements might be beneficial for the regulation of DNA condensation and gene expression during the transition from RB to EB.

Several phylogenetic trees have previously been constructed based on the *ompA *gene [14-17]. These trees separate the serovars into three groups: B complex (serovars B, Ba, D, E, L1 and L2), C complex (serovars A, C, H, I, Ia, J, K and L3) and the intermediate complex (serovars F and G). This classification does not represent biological differences in that both ocular strains and LGV strains are classified into the B and C complex. A phylogenetic analysis based on a concatenated nucleotide sequence from nine housekeeping genes, six intergenic non-coding segments and the *porB *gene gives a different classification in which the ocular and LGV strains are in separate clades [17]. That tree resembles the phylogenetic tree based on *hctB*, where the ocular strains are found in clade I and the LGV strains in clade V (Figure 3), thus it reflects the biological separation in distinct disease causing groups. Interestingly, both trees separate the reference strains for serotype D strains in the same way: D/UW-3 (10_DGHIIa) among serovar B (genital), G, H, I, Ia, J and K and D/IC-Cal8 (13_D) among serovar E and F.

The *hctB *gene with its high variability has proven to be a valuable target for discrimination between different *C. trachomatis *specimens in MLST analysis. For example, specimens with *ompA *genotype identical to the reference strain E/Bour constitute 37-45% in two major Swedish genotyping studies [18,19] and are abundant in the MLST database (allele number 1, 3-5, 7, 14, 21-25, 35 in Figure 3A). However, the *hctB *gene can discriminate these samples because of ten configurations of 4 and 5 elements in the repetitive region.

### Hc2 in *Chlamydiales spp*

**Figure 4:**
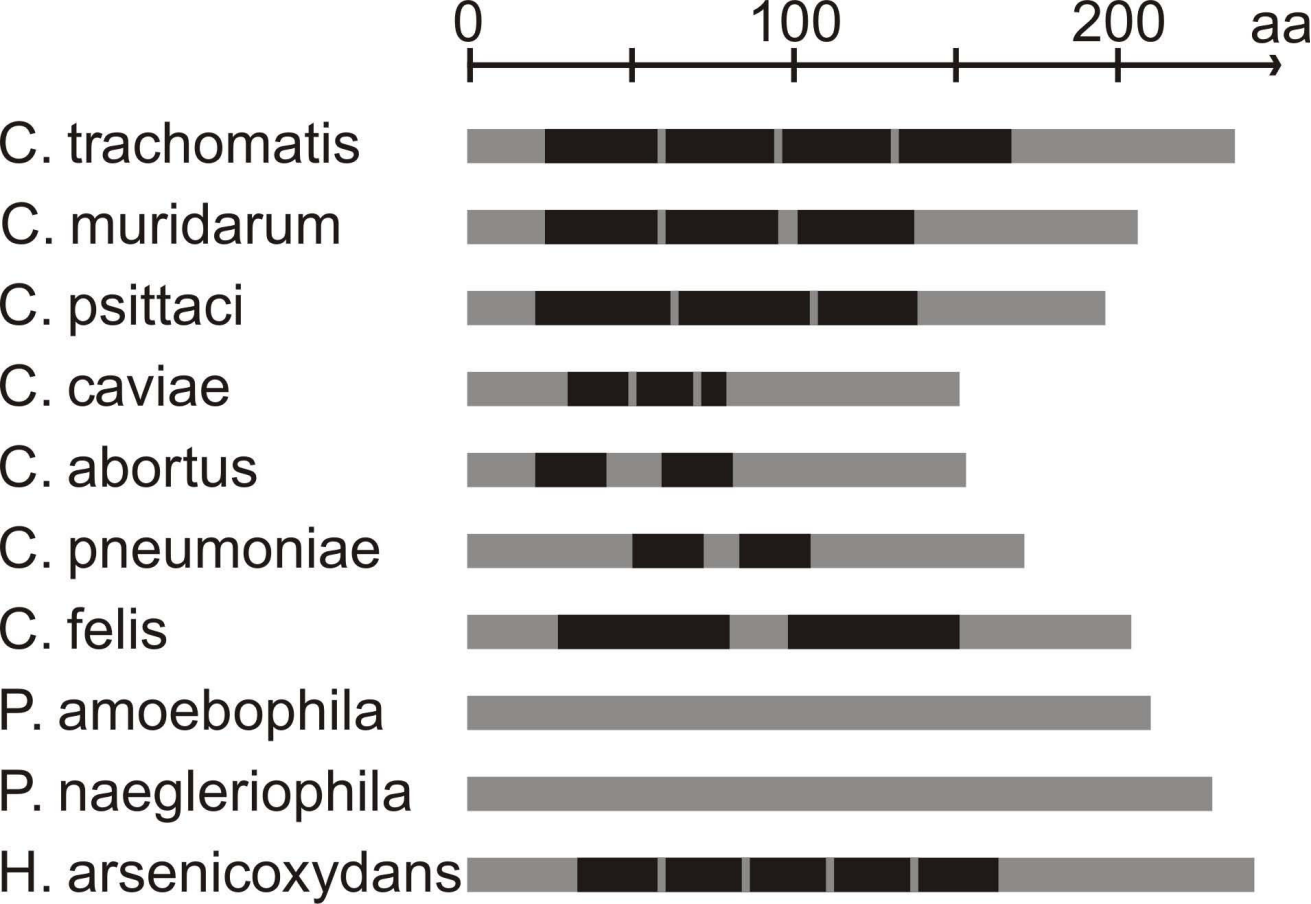
**Schematic overview of repetitive elements in Hc2 in the *Chlamydiales *order and in an Hc2-like protein in *Herminiimonas arsenicoxydans***. Repetitive elements of 20 amino acids or longer are shown in black.

Comparisons of *hctB *nucleotide sequences for other species in the *Chlamydiales*-order show that they have a similar structure with a region of repetitive elements built up by pentamers (Figure 4) and conserved flanking regions. The Hc2 sequence from the most closely-related species, *Chlamydia muridarum*, has the highest similarity to *C. trachomatis*, with three repetitive elements similar to the 1, 2 and 6 elements. The repetitive elements are shorter in *Chlamydophila abortus*, *Chlamydophila caviae *and *Chlamydophila pneumoniae *but longer in *Chlamydophila felis *and *Chlamydophila psittaci*. No repetitive elements were found in the more distantly related protochlamydial amoeba symbionts *Protochlamydia amoebophila *and *Protochlamydia naegleriophila*, and the pentameric structure was vaguer.

The *hctB *gene varies within *Chlamydophila abortus *and is one of the targets in a recently developed MLVA (multiple loci variable number of tandem repeat analysis) genotyping system [20]. In contrast, the four available genome sequences of *C. pneumoniae *are all identical in *hctB*. *C. pneumoniae *is difficult to differentiate with highly discriminatory methods (such as SNP analysis) [21] and is more conserved than *C. trachomatis *when using AFLP [22] or MLST [23].

### Hc2-like proteins in other genera

Searches in GenBank for Hc2-like proteins in other genera rendered hits including *Bordetella *(5 sequences), *Burkholderia *(31 sequences), *Herminiimonas *(1 sequence), *Minibacterium *(1 sequence) and *Ralstonia *(4 sequences). These proteins have a similar amino acid composition and similar pentamers, resulting in a distribution of positively charged residues almost identical to Hc2 (Figure 2).

These proteins vary both in length and repeat structure, and the rearrangement in the encoding genes might be as frequent as in *hctB *of *C. trachomatis. Burkholderia*, for instance, was found to have 14 size variants (149-231 amino acids) among 31 sequences from nine species. Longer repeats and several different kinds of repeats in the same protein were found in *Burkholderia ambifaria, Burkholderia cenocepacia, Burkholderia pseudomallei, Burkholderia vietnamensis *and *Burkholderia multivorans*. On the other hand, short consecutive repeats of only a pentamer were repeated seven and nine times in *Bordetella pertussis*, *Bordetella parapertussis *and *Bordetella bronchiseptica*. The Hc2-like proteins in *Bordetella petrii *and *Burkholderia phymatum *have no repeats. The protein most similar to Hc2 in *C. trachomatis *was found in *Herminiimonas arsenicoxydans *(Figure 4) and *Minibacterium masilliensis *with five and four repeats respectively.

Studies on the function of proteins similar to Hc2 have rarely been done in other genera. One exception is the BpH1 protein in *Bordetella *where consecutive lysine-rich pentamers causes size variation but which, unlike Hc2, is expressed during exponential growth and repressed in the stationary phase [24,25]. Strains with a knocked out *bpH1 *gene have a similar growth rate and phenotype as the wild-type strain, suggesting that this protein is not essential in *Bordetella*. No study on functional differences between strains with shorter or longer BpH1 has been conducted though BpH1 in *B. pertussis *has been reported to vary in size between 182 and 206 amino acids.

## Conclusions

To summarize, the size variation in Hc2 of *C. trachomatis *has previously been described as deletions of pentamers, but in the phylogenetic analyses we find a more complex evolutionary pattern of recurring nucleotide substitutions; deletions of elements and within-genome duplication of repeat elements. Our study shows that proteins similar to Hc2 also are present in several other bacterial groups. Phylogenetic analysis indicated that the corresponding *hctB *gene variants cluster in agreement with disease-causing properties. The high sequence variation of *hct*B provides a suitable target for genotyping of *C. trachomatis*. The essential DNA binding capacity of Hc2 in the unique biphasic life cycle of *Chlamydiacae *suggests that further experimental studies of DNA binding capacity and growth rate would provide increased knowledge about the role of length variation in Hc2.

## Methods

### Specimens and species

The MLST database [13] contained 378 sequences from clinical specimens or bacterial isolates (July 2009), of which 199 were from Sweden and the remaining 179 from Europe, Africa, North America and Australia. The strains included in the analysis are listed in additional file 1: appendix 1. The last 121 bp of the *hctB *gene are excluded from the MLST analysis. Consequently, additional sequencing was performed as previously described [11] but with the reverse primer hctB_R1 (5'-ATTTCGACTCAGCCAATAAATACA-3'). Sequences covering the *hctB *gene were aligned with ClustalW with default values in the BioEdit 7.0 sequence alignment editor (Ibis Therapeutics, Carlsbad, CA). The repetitive elements were aligned based on homology according to neighbour-joining phylogenetic analysis of the different types of repeat element. Obtained sequence variants were submitted to GenBank and the accession numbers are listed in additional file 2: appendix 2. Accession numbers for Hc2 in other *Chlamydiales *and Hc2-like proteins in other genera are listed in additional file 3: appendix 3.

### Sequence analysis

Repetitive amino acid elements were found with Dottup plots using a word size of 20 and Pepinfo was used to create plots that show the charge distribution. Both Dottup and Pepinfo are part of EMBOSS (The European Biology Open Software Suite, EMBnet, http://www.emboss.org).

### Phylogenetic analyses

Firstly, the phylogenetic relationship of the different types of repetitive element was estimated with a neighbour-joining analysis [26] based on the absolute number of base differences between the repeat element sequences (since this number is small, correction for multiple substitutions is not necessary). The resulting tree (Figure 3C) was used as the guide tree for manually adjusting the alignment of the repetitive elements (Figure 3B) in the alignment of the MLST sequences that include *hctB*.

Secondly, the phylogeny of the 41 variants of MLST targets was inferred using a Bayesian approach [e.g.', [27]]. The analysis was done with MrBayes 3.1.2, running under MPI [28]. A Bayesian analysis needs an explicit substitution model, and this was selected based on a hierarchical likelihood ratio test (ηLRT) approach [29] using Modeltest [30] together with PAUP* 4.0b10 [31].

MrBayes uses a Metropolis-coupled Markov chain Monte Carlo method to compute the posterior probabilities for the clades. This algorithm has no defined stop condition, but runs for a number of generations and must be monitored for convergence, and thus completion, of the algorithm. The convergence was assessed by monitoring the continuous-valued parameters using the software Tracer 1.4 [32], resulting in the Bayesian analysis being run for a total of 10^7 ^generations; the first 2.5 × 10^6 ^subsequently being discarded as burn-in (pre-convergence generations). The posterior probabilities were then summarized as a consensus tree with MrBayes.

Thirdly, the consensus tree was rooted by paralog rooting [33] based on the phylogeny of the repetitive elements from the first step, producing the final phylogenetic hypothesis.

Lastly, to check for conflicting signals and possible patterns of recombination, a recombination network of the sequences was computed using SplitsTree 4.10 [34].

## Authors' contributions

MK and BH designed the study and MK wrote the manuscript draft. MK and AN performed the sequence determination, analysed obtained sequences and identified the repetitive elements. MT performed the phylogenetic analyses. EBR analyzed protein strucures and together with MK found Hc2-like proteins in other genera. SB contributed intellectually since he has studied the Hc2 protein in the past. All authors participated in the writing process.

## Supplementary Material

Additional file 1 **Appendix 1**. List of the 378 sequences in the MLST database included in this study. Click here for file

Additional file 2 **Appendix 2**. Sequence variants of the MLST target that include *hctB *in *Chlamydia trachomatis *with corresponding accession number. Each sequence variant is named after the allele number and the serotypes in which that variant has been found. Click here for file

Additional file 3 **Appendix 3**. Hc2 amino acid sequences in *Chlamydiales *and Hc2-like sequences in other genera. Click here for file
