# Supplementary material for: Mosaic structure of intragenic repetitive elements in histone H1-like protein Hc2 varies within serovars of Chlamydia trachomatis
Source: BMC Microbiol. 2010 Mar 17;10:81. doi: 10.1186/1471-2180-10-81 (PMC2848022; doi:10.1186/1471-2180-10-81)
Supplement: Additional file 2 — Appendix 2. Sequence variants of the MLST target that include hctB in Chlamydia trachomatis with corresponding accession number. Each sequence variant is named after the allele number and the serotypes in which that variant has been found. [file 1471-2180-10-81-S2.DOC]

| ***hctB* variant** | **Accession number in GenBank** |
| --- | --- |
| 1_EF | GQ131810 |
| 2_Ba | GQ131811 |
| 3_E | GQ131812 |
| 4_E | GQ131813 |
| 5_DEF | GQ131814 |
| 6_G | GQ131815 |
| 7_DE | GQ131816 |
| 8_BGI | GQ131817 |
| 9_D | GQ131818 |
| 10_DGHIIaJK | GQ131819 |
| 11_BD | GQ131820 |
| 12_DHJK | GQ131821 |
| 13_D | GQ131822 |
| 14_E | GQ131823 |
| 15_C | GQ131824 |
| 16_AB | GQ131825 |
| 17_B | GQ131826 |
| 18_L1L2 | GQ131827 |
| 19_L3 | GQ131828 |
| 20_G | GQ131829 |
| 21_E | GQ131830 |
| 22_E | GQ131831 |
| 23_E | GQ131832 |
| 24_E | GQ131833 |
| 25_E | GQ131834 |
| 27_L2 | GQ131835 |
| 29_GJ | GQ131836 |
| 30_G | GQ131837 |
| 31_B | GQ131838 |
| 33_K | GQ131839 |
| 34_D | GQ131840 |
| 35_E | GQ131841 |
| 36_J | GQ131842 |
| 37_J | GQ131843 |
| 38_I | GQ131844 |
| 39_A | GQ131845 |
| 40_AB | GQ131846 |
| 41_B | GQ131847 |
| 42_K | GQ131848 |
| 43_F | GQ131849 |
| 44_L2 | GQ131850 |
