# Supplementary material for: Mosaic structure of intragenic repetitive elements in histone H1-like protein Hc2 varies within serovars of Chlamydia trachomatis
Source: BMC Microbiol. 2010 Mar 17;10:81. doi: 10.1186/1471-2180-10-81 (PMC2848022; doi:10.1186/1471-2180-10-81)
Supplement: Additional file 3 — Appendix 3. Hc2 amino acid sequences in Chlamydiales and Hc2-like sequences in other genera. [file 1471-2180-10-81-S3.DOC]

| **Genus** | **Species** | **Strain/ specimen** | **Locus tag** | **RefSeq Accession number** |
| --- | --- | --- | --- | --- |
| *Bordetella* | *avium* | 197N | BAV2984 | YP_787479 |
|  | *bronchiseptica* | RB50 | BB4378 | NP_890912 |
|  | *parapertussis* | 12822 | BPP3905 | NP_886055 |
|  | *pertussis* | Tohama1 | BP2985 | NP_881561 |
|  | *petrii* | DSM12804 | Bpet0581 | YP_001629184 |
| *Burkholderia* | *ambifaria* | AMMD | Bamb_0500 | YP_772393 |
|  | *ambifaria* | MEX-5 | BamMEX5DRAFT_3025 | ZP_02907671 |
|  | *ambifaria* | MC40-6 | BamMC406_0524 | YP_001807237 |
|  | *cenocepacia* | AU1054 | Bcen_0115 | YP_620002 |
|  | *cenocepacia* | HI2424 | Bcen2424_0598 | YP_834244 |
|  | *cenocepacia* | J2315 | BCAL3427 | YP_002232529 |
|  | *cenocepacia* | MC0-3 | Bcenmc03_0567 | YP_001763867 |
|  | *mallei* | ATCC10399 | BMA10399_J0061 | YP_002013978 |
|  | *mallei* | ATCC23344 | BMA2508 | YP_104054 |
|  | *mallei* | NCTC10229 | BMA10229_A1288 | YP_001027272 |
|  | *mallei* | NCTC10247 | BMA10247_3276 | YP_001082792 |
|  | *mallei* | FMH | BMAFMH_0760 | YP_002058543 |
|  | *mallei* | SAVP1 | BMASAVP1_A0429 | YP_991779 |
|  | *multivorans* | ATCC17616 | Bmul_2786 | YP_001580967 |
|  | *phymatum* | STM815 | Bphy_2636 | YP_001858854 |
|  | *phytofirmans* | PsJN | Bphyt_3433 | YP_001897047 |
|  | *pseudomallei* | 1106a | BURPS1106A_3510 | YP_001067747 |
|  | *pseudomallei* | 1655 | BURPS1655_E0309 | YP_002034810 |
|  | *pseudomallei* | 1710b | BURPS1710b_3509 | YP_334880 |
|  | *pseudomallei* | 668 | BURPS668_3472 | YP_001060483 |
|  | *pseudomallei* | 7894 | Bpse7_010100019606 | ZP_02483361 |
|  | *pseudomallei* | Pakistan 9 | Bpseu9_010100019592 | ZP_02457356 |
|  | *pseudomallei* | DM98 | BpseD_010100019956 | ZP_02404530 |
|  | *pseudomallei* | K96243 | BPSL2990 | YP_109584 |
|  | *pseudomallei* | NCTC13177 | BpseN_010100018976 | ZP_02491548 |
|  | *pseudomallei* | S13 | BURPSS13_U0090 | YP_002056051 |
|  | *thailandensis* | Bt4 | BthaB_010100020112 | ZP_02387255 |
|  | *thailandensis* | E264 | BTH_I1155 | YP_441702 |
|  | *thailandensis* | TXDOH | BthaT_010100020343 | ZP_02373390 |
|  | *vietnamiensis* | G4 | Bcep1808_0573 | YP_001118420 |
|  | *xenovorans* | LB400 | Bxe_A0525 | YP_560460 |
| *Chlamydia* | *muridarum* | Nigg | TC0316 | NP_296695 |
| *Chlamydophila* | *abortus* | S26/3 | CAB398 | YP_219812 |
|  | *caviae* | GPIC | CCA00412 | NP_829280 |
|  | *felis* | Fe/C-56 | CF0596 | YP_515512 |
|  | *pneumoniae* | AR39 | CP0371 | NP_444919 |
|  | *pneumoniae* | CWL029 | CPn0384 | NP_224584 |
|  | *pneumoniae* | J138 | CPj0384 | NP_300441 |
|  | *pneumoniae* | TW-183 | CpB0396 | NP_876670 |
| *Herminiimonas* | *arsenicoxydans* | ULPAs1 | HEAR2768 | YP_001101006 |
| *Minibacterium* | *massiliensis* | Marseille | mma_2977 | YP_001354667 |
| *Protochlamydia* | *amoebophila* | UWE25 | pc1836 | YP_008835 |
| *Ralstonia* | *eutropha* | H16 | H16_A3178 | YP_727621 |
|  | *solanacearum* | GMI1000 | RSc2793 | NP_520914 |
|  | *solanacearum* | MolK2 | RSMK01673 | YP_002251894 |
|  | *solanacearum* | UW551 | RRSL_02664 | ZP_00943850 |
